# A randomised, double blind, placebo-controlled trial of megestrol acetate or dexamethasone in treating symptomatic anorexia in people with advanced cancer

**DOI:** 10.1038/s41598-021-82120-8

**Published:** 2021-01-28

**Authors:** David C. Currow, Paul Glare, Sandra Louw, Peter Martin, Katherine Clark, Belinda Fazekas, Meera R. Agar

**Affiliations:** 1 IMPACCT, Faculty of Health, University of Technology Sydney, P O Box 123, Ultimo, NSW 2007 Australia; 2 Pain Management Research Institute, Northern Clinical School, University of Sydney, St Leonard’s, NSW Australia; 3 McCloud Consulting Group, Belrose, NSW Australia; 4 Clinical Communication and End-of-Life Care, School of Medicine, Deakin University, Melbourne, Australia; 5 Department of Palliative Care, Calvary Mater, Newcastle, NSW Australia; 6 School of Medicine and Public Health, The University of Newcastle, Newcastle, NSW Australia; 7 College of Medicine and Public Health, Flinders University, Adelaide, SA Australia

**Keywords:** Cancer, Drug delivery

## Abstract

This multi-site, double blind, parallel arm, fixed dose, randomised placebo controlled phase III study compared megestrol acetate 480 mg/day with dexamethasone 4 mg/day for their net effects on appetite in people with cancer anorexia. Patients with advanced cancer and anorexia for ≥ 2 weeks with a score ≤ 4 (0–10 numeric rating scale (NRS) 0 = no appetite, 10 = best possible appetite) were recruited. Participants received megestrol 480 mg or dexamethasone 4 mg or placebo daily for up to 4 weeks. Primary outcomes were at day 7. Responders were defined as having a ≥ 25% improvement in NRS over baseline. There were 190 people randomised (megestrol acetate n = 61; dexamethasone n = 67, placebo n = 62). At week 1 (primary endpoint), 79·3% in the megestrol group, 65·5% in the dexamethasone group and 58·5% in the placebo group (*p* = 0.067) were responders. No differences in performance status or quality of life were reported. Treatment emergent adverse events were frequent (90·4% of participants), and included altered mood and insomnia. Hyperglycemia and deep vein thromboses were more frequent when on dexamethasone than the other two arms. There was no difference in groups between the three arms, with no benefit seen over placebo with anorexia improving in all arms.

Trail registration: The trial was registered on 19/08/2008 with the Australian New Zealand Clinical Trials Registry (ACTRN12608000405314).

## Introduction

Anorexia, the loss of appetite, occurs in 30–80% of people with cancer referred to palliative care^1^. It contributes to impaired quality of life (QoL), increased morbidity and mortality^2^. Anorexia is a challenging symptom to study, given its subjective nature, its fluctuation and the lack of definition of ‘normal’ appetite for the population generally, let alone people with advanced disease. Thus, there are no established thresholds to determine at which point a person is considered to have anorexia, nor a critical duration of time before it is considered clinically concerning^3,4^. Often as part of a pro-inflammatory state, anorexia is associated with raised levels of interleukin-6 (IL-6) and C-reactive protein (CRP) in people with cancer^5^.

Although there has been progress in better defining and understanding cachexia in recent years^6^ as part of the cancer anorexia cachexia syndrome (CACS), this current study deals specifically with the symptom of loss of appetite and its impact on health outcomes including health-related QoL^7–9^. Symptomatic treatment of anorexia (which is unlikely to have any effect on cachexia given that cachexia per se does not respond to nutritional support) remains an unmet clinical need with a number of pharmacological interventions used, but no clear standard of care given inconsistent effects on appetite and quality of life^10–12^. Importantly, response on one disease state to a symptomatic therapy for anorexia does not necessarily translate to benefit for people with cancer^13^. Goals of therapy for anorexia include maintenance or improvement of appetite while optimising QoL.

Late in life, anorexia can cause significant distress to patients^14^. Even with counselling, anorexia is also often distressing to families and caregivers, and their perspectives are important when considering interventions^4,15,16^. In palliating anorexia in people with advanced cancer, an approach which focuses on stimulating appetite, supporting each person’s food preferences, avoiding prescriptive dietary advice and providing education to patients and their caregivers, are crucial. This education is to promote a shared understanding about changed goals of care and to help to reduce the distress caused by reduced oral intake.

Recommended approaches to appetite stimulation in patients undergoing palliative care for cancer include megestrol or dexamethasone^8^. Landmark studies have been done in megestrol, but these have not included a dexamethasone arm (the most frequently prescribed medication for anorexia in this setting) or a placebo arm, and have not had sufficient evidence to make a health economic case for its use (despite the fact that it is off-patent)^17,18^. These studies were brought together in three systematic reviews over the last decade^19–21^. There is one seminal, placebo-controlled randomised trial on dexamethasone for anorexia in which improved appetite was seen in the two dexamethasone groups, but with short lived benefits and no improvement in weight or performance status, and no difference in survival^22^. Most recently, the European Society for Clinical Nutrition and Metabolism (ESPEN) has put forward evidence-based guidelines for nutritional assessment and interventions in people with advanced cancer, noting that nutrition has a role in the setting of malnutrition if that is present in people with advanced cancer^23^.

The study brings together the two most frequently used medications for anorexia in advanced cancer and for the first time includes a placebo arm in their comparison and prospective data collection to inform a health economic evaluation. There are concerns about the unwanted side effects of both medications. Potentially, these side effects may be amplified in advanced cancer and any cost–benefit relationship negated. Other drugs that have been trialed but are not in widespread use to treat anorexia in advanced cancer include medroxyprogesterone, pentoxifylline, anabolic steroids, cannabinoids, mirtazapine and olanzapine^24–30^. Despite evidence supporting the use of megestrol for anorexia^20,21^, it is not currently prescribed extensively as an appetite stimulant to people with advanced cancers, partially due to its cost (despite being off patent, so many jurisdictions do not reimburse for the indication of anorexia), and partially because there is a lack of clinical consensus on the optimal dose, frequency and treatment duration in this population^24,31^.

The aim of this phase III study was to compare megestrol acetate 480 mg/day, dexamethasone 4 mg/day and placebo for their net effect on short-term appetite (not cachexia) and QoL in people with advanced cancer. The seven day duration was chosen, because if no benefit is seen in that time in this population, it is unlikely to have wide clinical uptake.

## Participants and methods

### Participants

Participants were recruited from 12 centres covering 23 institutions across Australia. Patients were included if they were: aged ≥ 18 years; diagnosed with advanced cancer and were known to a palliative care team; were mentally competent; able to take oral medications; had a baseline appetite score of ≤ 4 on a 0–10 numeric rating scale (NRS; where 0 is no appetite and 10 is best possible appetite); and an Eastern Cooperative Oncology Group (ECOG) score of 0–3 or Australia-modified Karnofsky performance status (AKPS) score of 30–100^32,33^. Participants were excluded if they: had a history of documented thromboembolic disease or an implanted vascular access device without adequate anticoagulation using local protocols; had severe or uncontrolled ischemic heart disease, congestive cardiac failure or severe hypertension (systolic blood pressure > 180 mmHg); were taking corticosteroids (excluding inhaled corticosteroids) or progestogens; were tube fed; had clinically significant ascites (given theoretical concerns of worsening fluid retention with the active interventions); had unmonitored diabetes; had uncontrolled nausea and vomiting; were pregnant or breastfeeding; had an active infection; or had diarrhea within the last 7 days. All participants and, separately, their caregivers provided written informed consent.

### Trial design

This study was conducted in accordance with International Committee on Harmonization—Good Clinical Practice (ICH GCP)^34^. The study was approved by the respective human research ethics committees at each participating site. (Table 1) Participants were randomized 1:1:1 to receive megestrol acetate 480 mg (three capsules of 160 mg megestrol), dexamethasone 4 mg (one capsule containing 4 mg dexamethasone and two placebo capsules) or placebo (three capsules) daily for up to four weeks. Randomisation schedules were developed for each site using random number tables generated at an independent site using block sizes of four. The dispensing pharmacist allocated a sequential code according to the supplied schedule. Capsules were prepared in a central manufacturing pharmacy, where tablets were encased in an opaque capsule to have all study drugs and placebo appearing identical. All study staff, along with participants were blinded to the treatment allocation for the entire duration of the study.

**Table 1:** Full names of ethics committees that approved this study.

Ethics committee	Recruitment site	Ethics approval date
Mater Health Services Human Ethics Research Committee	Mater Health Services, QLD	03/10/2008
Peter MacCallum Research Centre	Peter MacCallum Cancer Centre, VIC	08/01/2009
Flinders Clinical Research Ethics Committee	Flinders Medical Centre, SA	10/02/2009
Hollywood Private Hospital Research Ethics committee	Hollywood Private Hospital, WA	03/03/2009
Ballarat Health Services SJOGHC Human Research Ethics Committee	Ballarat Health Services, VIC	01/05/2009
Sydney South West Human Research Ethics Committee (RPAH Zone)	Braeside Hospital, NSW	09/02/2009
Sydney South West Human Research Ethics Committee (RPAH Zone)	Camden Hospital, NSW	09/02/2009
Sydney South West Human Research Ethics Committee (RPAH Zone)	Calvary Mater Newcastle, NSW	09/02/2009
Sydney South West Human Research Ethics Committee (RPAH Zone)	Liverpool Hospital, NSW	09/02/2009
Flinders Clinical Research Ethics Committee	Flinders Private Hospital, NSW	05/05/2009
Flinders Clinical Research Ethics Committee	Blackwood Hospital, SA	07/05/2009
Sydney South West Human Research Ethics Committee (RPAH Zone)	Sacred Heart hospice, NSW	09/02/2009
St Vincent’s Health Human Research Ethics Committee-D	Caritas Christi Hospice, VIC	24/09/2009
St Vincent’s Health Human Research Ethics Committee-D	St Vincent’s Hospital Melbourne, VIC	24/09/2009
Alfred Hospital Ethics Committee	The Alfred Hospital, VIC	04/12/2009
Ballarat Health Services and St John of God Health Care HREC	St John of God Health Care Ballarat, VIC	25/03/2009
	Ballarat Health Services, VIC	
St John of God Health Care Ethics Committee	St John of God Hospital Murdoch, WA	08/04/2010
	St John of God Health Care Subiaco, WA	
Barwon Health Human Research Ethics Committee	Barwon Health Palliative Care Program Geelong , VIC	03/06/2010
	Barwon Health Palliative Care Program McKellar Centre, VIC	03/06/2010
Alfred Hospital Ethics Committee	Caulfield Hospital, VIC	15/12/2010
Hammondcare Health and Hospitals	Greenwich Hospital, Hammond Care, NSW	25/09/2012

**Figure 1:**
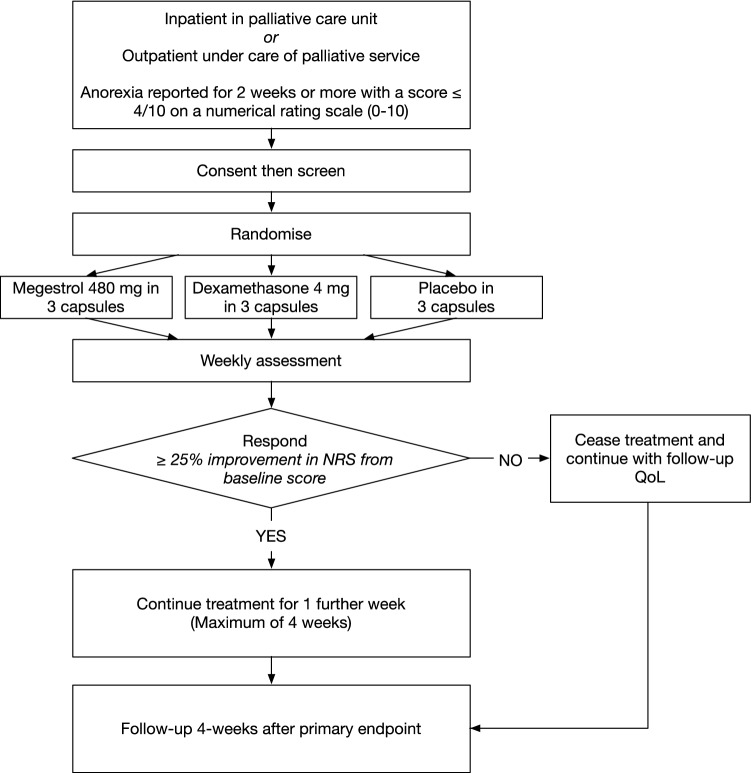
Study diagram for the multi-site, parallel arm, fixed dose, randomised controlled trial of 190 participants with cancer and anorexia comparing megestrol or dexamethasone with placebo.

Participants attended a screening visit, a baseline visit, then weekly visits for up to four weeks. A follow-up visit was conducted one week after treatment cessation (Fig. 1).

During the four-week treatment period, participants received morning doses of their allocated intervention. They underwent weekly assessments of blood glucose levels, AKPS^32^, ECOG^33^, weight, and Functional Assessment of Anorexia/ Cachexia Therapy (FAACT)^35^, Memorial Symptom Assessment Scales (MSAS)^36^, efficacy, treatment adherence, adverse events, and concomitant medication review. If participants had not responded to the allocated treatment by the end of each treatment week, they ceased randomised treatment and attended a study completion visit.

The primary outcome was the change from baseline in appetite score on a NRS at the end of Week 1. Participants were considered to be responders if they had a 25% improvement over the baseline score or a one point improvement if the baseline score was zero. This is a conservative estimate of the minimum clinically important difference in improvement in appetite scores which may be as low as 0.6 points on a 0–10 NRS^37^. Secondary outcomes included weight, performance status (AKPS)^32^, appetite score using the single appetite distress item from the MSAS^36^ and the ‘Additional Concerns’ section of the FAACT questionnaire^35^; change in general health related QoL (Functional Assessment of Cancer Therapy—General; FACT-G)^38^; and adverse events. An Independent Data Safety Monitoring Committee (DSMC) monitored adverse events 6 monthly, and ad hoc as required. Data were collected prospectively for a health economic analysis and will be reported separately.

### Statistical plan

The primary objective was to compare response rates between arms. For there to be a clinically meaningful difference between arms (accounting for known harm rates for these medications), it was assumed that the background rate would be 20% and there would need to be a difference of 25% between each treatment group and the placebo group. Based on previous data, a response rate of 45% in each treatment group was therefore assumed. This meant a sample size of 53 participants per group (159 in total) provided 80% power with a two-tailed alpha set at 0·05. Analyses were conducted on an intention-to-treat (ITT) basis. Pairwise comparisons (megestrol versus placebo; and dexamethasone versus placebo) were conducted using Chi-square tests.

In order to control for multiplicity in the primary endpoint, first the global test of association between treatment group and the primary endpoint was assessed at the 5% significance level. Only if the global test of association was significant were the pairwise treatment comparisons tested at the 5% significance level. Secondary outcomes were analysed using the same approach. Changes in weight, AKPS and MSAS appetite scores between the placebo group and the megestrol and dexamethasone groups were examined using Mann Whitney U tests. Quality of life was analysed using analysis of covariance (ANCOVA) with the baseline result as a covariate, and centre and treatment group as predictor variables as was caregiver wellbeing. Adverse events were compared using Mann–Whitney U tests.

In all change-from-baseline analyses, baseline values were used as covariates. No imputation of missing data was performed given the weekly review of response for continued participation in the study. Data for participants who withdrew early from the study are included up to the point of their discontinuation. Data are presented as n (%) or mean (standard deviation (SD)), unless otherwise noted. The study is reported using the CONSORT guidance for randomized, controlled trials^39^.

### Ethics approval and consent to participate

The study was approved by the respective human research ethics committees at each participating site.

## Results

### Participant characteristics

**Figure 2:**
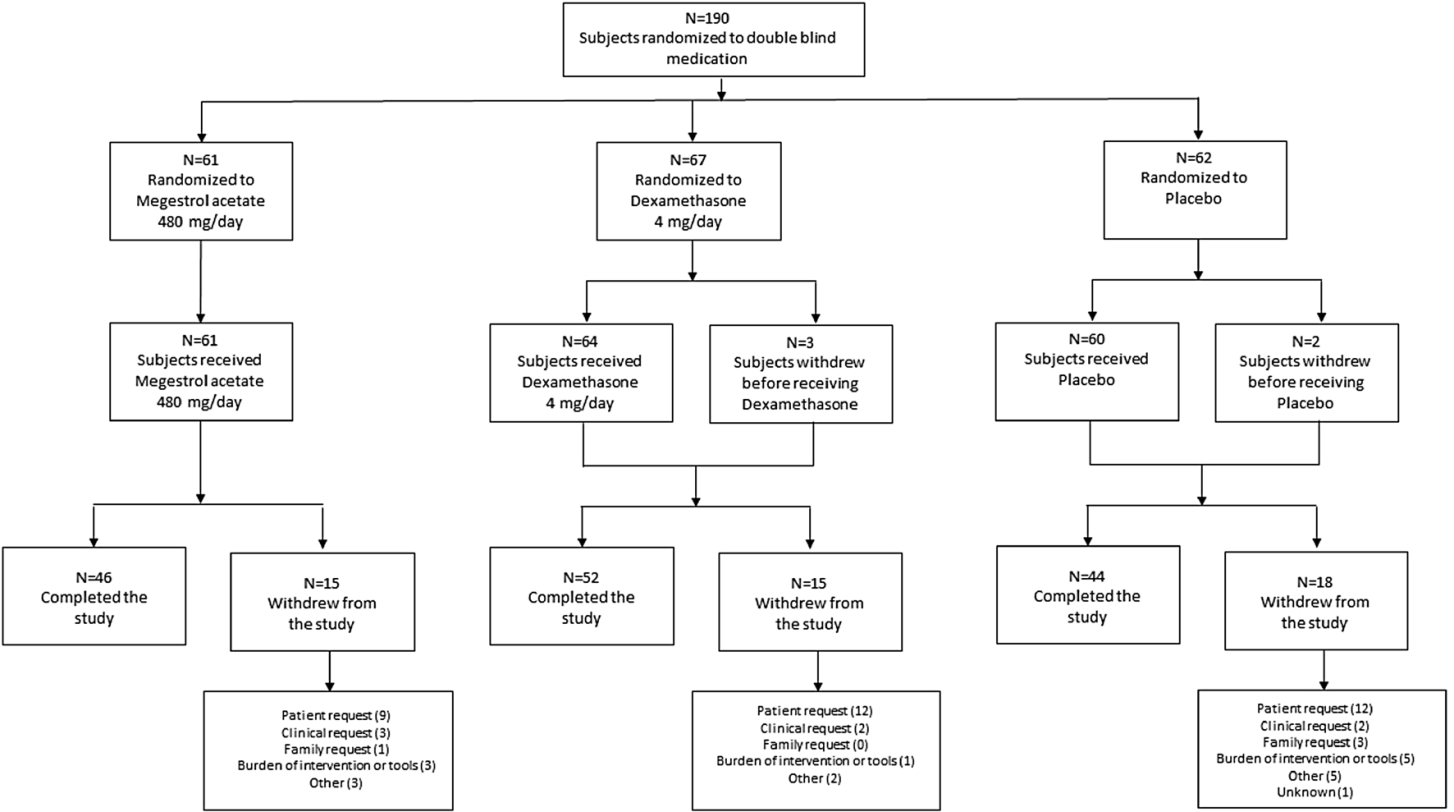
CONSORT diagram of a multi-site, parallel arm, fixed dose, randomised controlled trial of 190 participants with cancer and anorexia comparing megestrol or dexamethasone with placebo.

From January 2009 to March 2015, 190 participants typical of a palliative care population (Table 2) with anorexia for at least the preceding two weeks were randomised to double-blind treatment (megestrol acetate n = 61; dexamethasone n = 67, placebo n = 62; Fig. 2). All participants had advanced, progressive cancer: lung cancer was the most prevalent underlying diagnosis (n = 56, 29·5%) followed by colorectal cancer (n = 25, 13·2%), prostate cancer (n = 23, 12·1%) and other gastrointestinal cancers (n = 20, 10·5%; Table 2). Treatment groups were well balanced for demographics and clinical baseline characteristics. The major reason for screen failure was the number of people already on a long-term corticosteroid.

**Table 2:** Participant demographics at baseline in a multi-site, parallel arm, fixed dose, randomised controlled trial of 190 participants with cancer and anorexia comparing megestrol or dexamethasone with placebo.

	Megestrol acetate 480 mg/day (N = 61)	Dexamethasone 4 mg/day (N = 67)	Placebo (N = 62)
Age at screening, years, mean (SD)	71.4 (10.9)	71.2 (12.7)	74.7 (10.0)
**Gender, n (%)**			
Male	36 (59.0%)	39 (58.2%)	39 (62.9%)
Female	25 (41.0%)	28 (41.8%)	23 (37.1%)
Weight, kg, mean (SD)	63.1 (14.0)	62.5 (14.6)	62.9 (13.9)
	n = 58	n = 64	n = 58
Height, cm, mean (SD)	167.6 (9.9)	167.1 (10.2)	168.2 (10.2)
	n = 57	n = 60	n = 57
BMI, kg/m^2^, mean (SD)	22.36 (3.95)	21.97 (4.00)	22.22 (3.55)
	n = 55	n = 60	n = 56
**ECOG performance status**			
0	0 (0.0%)	1 (1.5%)	1 (1.6%)
1	15 (24.6%)	13 (19.4%)	18 (29.0%)
2	29 (47.5%)	29 (43.3%)	31 (50.0%)
3	16 (26.2%)	24 (35.8%)	12 (19.4%)
4	1 (1.6%)	0 (0.0%)	0 (0.0%)
AKPS score, median (min, max)	70 (30, 90)	60 (20, 90)	70 (20, 90)
Baseline NRS for appetite, median (min, max)	3 (0, 4)	2 (0, 4)	3 (0, 4)

*AKPS* Australia-modified Karnofsky performance scale, *BMI* body mass index, *ECOG* Eastern Co-operative Oncology Group, *NRS* numerical rating scale.

### Treatment exposure

The mean days of exposure to treatment was greatest in the megestrol group (17.4 (SD 9.0) days) compared to dexamethasone (15.7 (SD 9.7) days) or placebo (11.7 (SD 8.9) days) predominantly reflecting the planned withdrawal from treatment if insufficient response was obtained at the end of each study week. (Supplementary Fig. 1) More than half of participants in the placebo group (56.7%) had seven or fewer days of treatment, compared to 21.3% in the megestrol arm, and 35.9% in the dexamethasone arm.

### Primary endpoint*: *NRS appetite response

At week 1 (primary endpoint), 79.3% of participants in the megestrol group, 65.5% in the dexamethasone group and 58.5% in the placebo group were responders (Table 3). The overall association between treatment group and NRS appetite response was not statistically significant (*p* = 0.067, Wald type 3 Chi-Square for test of treatment association), and thus the primary endpoint of the study was not met. The overall pattern of response week by week by group is shown in Supplementary Fig. 2. Therefore, pairwise comparisons were not tested. Overall, the odds of a response compared to placebo were 2.68 (95% CI 1.15–6.23) for participants in the megestrol group, and 1.34 (95% CI 0.62–2.90) for participants in the dexamethasone group. The high rates of response in all arms in weeks two to four reflects protocol-driven withdrawal of participants who failed to achieve an adequate symptomatic response at the end of each treatment period.

**Table 3:** Proportion of numerical rating scale (NRS) appetite responders by arm at week 1 (the primary end-point) and the scores of those who subsequently continued because of their response in week 1.

	Megestrol acetate 480 mg		Dexamethasone 4 mg		Placebo	
	%	N assessable	%	N assessable	%	N assessable
Week 1	79.3	58	65.5	58	58.5	53
Week 2^a^	92.5	40	96.9	32	78.9	19
Week 3^a^	89.7	29	100.0	28	100.0	12
Week 4^a^	100.0	22	100.0	20	100.0	10
Follow-up	88.5	52	81.8	44	52.4	42

^a^Note participants that failed to achieve a response at the end of each treatment period ceased treatment and underwent a follow-up visit.

### Secondary endpoints: *Weight*

Weight was considered stable if there was a ≤ 1 kg reduction from baseline. There was no differences in weight stability between groups (*p* = 0.2417), and therefore pairwise comparisons were not conducted.

### Change in MSAS appetite scores

Another appetite response was defined as a reduction from baseline of one or more distress category on the MSAS. Overall, treatment had a significant effect on MSAS appetite response rates at week 1 (68.2% in megestrol group, 38.3% in the dexamethasone group and 48.9% in the placebo group, *p* = 0.0162), however the pairwise comparisons with placebo were not significant (for megestrol/placebo (*p* = 0.0697) nor dexamethasone / placebo (*p* = 0.3114). There was no difference in the proportion of responders on the FAACT anorexia sub-scale.

### Quality of life

In participants, there was no difference in QoL from baseline to week 1 in the FACT-G total score), nor any of the domain scores. There were no differences in caregivers’ QoL (CQOLC; Table 4).

**Table 4:** Secondary endpoints including weight and appetite at week 1 of a multi-site, parallel arm, fixed dose, randomised controlled trial of 190 participants with cancer and anorexia comparing megestrol or dexamethasone with placebo.

	Megestrol acetate 480 mg *p* value compared to placebo	Dexamethasone 4 mg *p* value compared to placebo	Placebo	Overall *p* value
Weight responders, %	87%	74%	85%	0.2417
MSAS^ responders, %	68.2% *p* = 0.0697	38.3% *p* = 0.3114	48.9%	0.0162
AKPS^^ responders, %	98%	95%	92%	0.3367
Change from baseline in FACT-G* Total Score, mean (SD)	− 2.1 (3.4)	− 4.8 (3.4)	− 0.8 (3.4)	0.576
CQOLC** score, mean (SE)	89.6 (7.1)	79.8 (6.5)	81.2 (7.9)	0.520

Other than hyperglycaemia which was higher in the dexamethasone arm, rates of events actively sought were similar between groups.

^MSAS—Memorial Symptom Assessment Scale; ^^AKPS—Australia-modified Karnofsky Performance Status scale; *FACT-G: Functional Assessment Cachexia Therapy—General; **CQOLC: Caregiver Quality of Life Index—Cancer.

### Other measures

There was no association between treatment group and AKPS maintenance at week 1 (Table 4).

### Adverse events

Almost all participants experienced at least one adverse event (91·4% in megestrol arm, 89.1% in dexamethasone arm, and 91.7% in the placebo arm). Common Terminology Criteria Grade 3, 4 or 5 adverse events of special interest occurred in less than 30% of participants (Table 5). In general, the profile of treatment emergent adverse events (TEAE) was similar between all arms. TEAEs of particular interest experienced by most participants were: altered mood (31.1%); insomnia (27.9%); and muscular weakness (27.9%) in the megestrol group: insomnia (39.1%); hyperglycaemia (32.8%); and altered mood (31.3%) in the dexamethasone group: and muscular weakness (35.0%); oedema (30.0%); altered mood (26.7%); and bone pain (26.7%) in the placebo group. There was no statistically significant association between treatment groups and the proportion of participants with at least one TEAE of special interest of any grade (*p* = 0.4346), however there was a significant association between hyperglycaemia of any grade and treatment group, with those in the dexamethasone group experiencing this event more frequently (32.8%) than either of the other treatment groups (megestrol 16·4% and placebo 11.7%). The differences between dexamethasone and placebo were statistically significant (*p* = 0.0037).

**Table 5:** Grade 3, 4 or 5 treatment emergent adverse events of special interest in a multi-site, parallel arm, fixed dose, randomised controlled trial of 190 participants with cancer and anorexia comparing megestrol or dexamethasone with placebo.

System organ class/preferred term	Megestrol Acetate 480 mg/day (N = 61) n (%)	Dexamethasone 4 mg/day (N = 64) n (%)	Placebo (N = 60) n (%)
Participants with at least one grade 3, 4 or 5 treatment emergent adverse event	10 (16.4%)	19 (29.7%)	12 (20.0%)
Gastrointestinal disorders	0 (0.0%)	1 (1.6%)	0 (0.0%)
Dyspepsia	0 (0.0%)	1 (1.6%)	0 (0.0%)
General disorders and administration site conditions	0 (0.0%)	1 (1.6%)	2 (3.3%)
Oedema	0 (0.0%)	1 (1.6%)	2 (3.3%)
Metabolism and nutrition disorders	2 (3.3%)	7 (10.9%)	1 (1.7%)
Hyperglycaemia	1 (1.6%)	7 (10.9%)	1 (1.7%)
Hyperkalaemia	1 (1.6%)	0 (0.0%)	0 (0.0%)
Musculoskeletal and connective tissue disorders	3 (4.9%)	3 (4.7%)	3 (5.0%)
Bone pain	1 (1.6%)	1 (1.6%)	1 (1.7%)
Muscular weakness	1 (1.6%)	2 (3.1%)	2 (3.3%)
Musculoskeletal disorder	0 (0.0%)	1 (1.6%)	0 (0.0%)
Osteonecrosis	1 (1.6%)	0 (0.0%)	0 (0.0%)
Psychiatric disorders	3 (4.9%)	2 (3.1%)	3 (5.0%)
Confusional state	1 (1.6%)	1 (1.6%)	3 (5.0%)
Insomnia	2 (3.3%)	1 (1.6%)	0 (0.0%)
Mood altered	1 (1.6%)	0 (0.0%)	0 (0.0%)
Renal and urinary disorders	0 (0.0%)	1 (1.6%)	0 (0.0%)
Acute kidney injury	0 (0.0%)	1 (1.6%)	0 (0.0%)
Respiratory, thoracic and mediastinal disorders	0 (0.0%)	1 (1.6%)	1 (1.7%)
Pulmonary embolism	0 (0.0%)	1 (1.6%)	0 (0.0%)
Pulmonary oedema	0 (0.0%)	0 (0.0%)	1 (1.7%)
Vascular disorders	3 (4.9%)	6 (9.4%)	3 (5.0%)
Deep vein thrombosis	2 (3.3%)	3 (4.7%)	0 (0.0%)
Hypertension	1 (1.6%)	3 (4.7%)	2 (3.3%)
Lymphoedema	0 (0.0%)	0 (0.0%)	1 (1.7%)

Other than hyperglycaemia which was higher in dexamethasone, rates of events actively sought were similar between groups.

Serious TEAEs occurred in 19 (31.1%), 19 (29.7%) and 20 (33.3%) of participants in the megestrol, dexamethasone and placebo arms, respectively. With megestrol, the serious TEAEs experienced were disease progression (4.9%), deep vein thrombosis (3.3%), general physical health deterioration (3·3%), and sepsis (3·3%). With dexamethasone, the most common were deep vein thrombosis (6.3%), lower respiratory tract infection (3.1%), urinary tract infection (3·1%), rectal haemorrhage (3.1%), and pathological fracture (3.1%). In the placebo group, the most common were disease progression (8·3%), pleural effusion (5%), breathlessness (3·3%), dehydration (3·3%), and pneumothorax (3·3%).

There were 10 deaths recorded during the study, and all but two occurred during follow-up. All deaths were due to progressive disease although two were considered ‘unexpected’.

## Discussion

Overall, there were no statistically significant differences in the treatment effect of megestrol acetate 480 mg or dexamethasone 4 mg compared to placebo daily on any of the efficacy endpoints at week 1. The safety profiles of megestrol, dexamethasone and placebo were generally similar. As would be expected, participants treated with dexamethasone were more likely to experience hyperglycemia.

Other studies of megestrol and dexamethasone for the treatment of cancer-related anorexia have reported similar degrees of appetite stimulation in participants treated with dexamethasone 0·75 mg four times daily compared to megestrol acetate 800 mg daily, again with non-significant findings, but as with the current study the direction of benefit was in favour of megestrol^24^, as was one systematic review^40^.

The inclusion of a placebo arm in this study is an important advance on previous head-to-head comparisons or dose ranging studies. With the placebo arm, the natural history of the variability in NRS appetite scores is demonstrated, and no therapy was consistently superior to placebo. Any benefit attributed to one of the study medications is likely to be marginal given such a high rate of appetitie improvement in the placebo arm and needs to be weighted closely against the directly attributable harms from the intervention medications.

TEAEs remain a problem in participants with late-stage cancer in effectiveness studies. Of particular concern are mood alterations and hyperglycaemia, which have the potential to reduce participants’ QoL. It has been suggested that fatigue, anorexia-cachexia and depression may share a common pathophysiologic basis^41^, and therefore it is important to address all symptoms in order to improve QoL. Given the potential for dexamethasone to reduce cancer-related fatigue, it provides a possible mechanism for improvements in anorexia in participants administered dexamethasone at the significant cost of accelerating catabolic effects on muscle^42^. Concerns of high rates of thromboembolic disease in people in the megestrol arm were not reflected in the current results, with higher rates seen in the dexamethasone arm.

We have previously reported the unblinded rates of dexamethasone stimulating appetite in people with advanced life-limiting illnesses, however the data raised concerns about the potential for harms, particularly insomnia, psychotropic effects (depression, euphoria) and hyperglycaemia^43^.

The study took a long time to accrue despite being a multi-site study that engaged with trial-active clinicians. The major reason for problems in recruiting was the widespread use of dexamethasone, often with little information in the clinical notes as to why it had been commenced and, importantly, why it was being continued.

The present study is limited by its design, which required participants with insufficient response to cease randomised treatment. Therefore, only results at week 1 are reliable for attributing changes in NRS and MSAS appetite scores to the arm to which participants were randomised. Like all studies of interventions for subjective symptom management, the response seen in the placebo arm includes regression to the mean, natural variability in the symptom and a placebo effect^44^, potentially obscuring the effect of the pharmacologically active substances under evaluation. The only way to overcome this methodological conundrum would be to use complex study designs with multiple arms that include “balanced placebos” with the active substances deceptively labeled as “placebo” and placebos labeled as “active”^45^. This study was exploring anorexia only, so measures specific to cachexia such as body composition were not undertaken.

The other fundamental challenge was the choice of dose in a study with two active arms. Both medications have a dose–response effect, so choice for each when compared to placebo, and especially when compared to each other becomes very important in interpreting the results. The exact dose equivalence between dexamethasone and megestrol *for appetite* has not been defined.

There are currently a number of ongoing studies of dexamethasone or megestrol, including a phase III trial comparing levocarnitine, celecoxib, carbocisteine and megestrol for increasing lean body mass in people with advanced malignancy (EUCTR2010-019831-36-IT). A pilot study of megestrol in combination with formoterol in participants with advanced malignancy (EUCTR2008-008857-53-GB) showed promising results for the combination of an anabolic β2-agonist and megestrol to stimulate appetite^46^. A phase III evaluation of these findings is of great interest to the clinical and scientific community.

This study can inform future studies. In a similar study in the future, an absolute, rather than a relative improvement should be used. Given the low rates of thromboembolic events in the megestrol arm, it would be reasonable to extend the time until the primary end point to two weeks.

## Conclusion

There was little difference between treatment groups in terms of the primary and secondary efficacy endpoints. This study does not establish a new standard of care for the symptomatic improvement of anorexia in people with advanced cancer.

## Supplementary Information

Supplementary Figures.

## Data Availability

Data are available on request from bona fide researchers.

## Code availability

Custom code is also available to bona fide researchers.
