# Supplementary material for: A randomised, double blind, placebo-controlled trial of megestrol acetate or dexamethasone in treating symptomatic anorexia in people with advanced cancer
Source: Sci Rep. 2021 Jan 28;11:2421. doi: 10.1038/s41598-021-82120-8 (PMC7844230; doi:10.1038/s41598-021-82120-8)
Supplement: Supplementary file 1 — Supplementary Figures. [file 41598_2021_82120_MOESM1_ESM.docx]

**A randomised, double blind, placebo-controlled trial of megestrol acetate or dexamethasone in treating symptomatic anorexia in people with advanced cancer**

David C. Currow ^1,7^

Paul Glare^2^

Sandra Louw^3^

Peter Martin^4^

Katherine Clark^5,6^

Belinda Fazekas^1,7^

Meera R. Agar^1^

^1^ IMPACCT, Faculty of Health, University of Technology Sydney, Ultimo, New South Wales, Australia.

^2^ Pain Management Research Institute, Northern Clinical School, University of Sydney. St Leonard’s, New South Wales, Australia.

^3^McCloud Consulting Group, Belrose, NSW, Australia

^4^ Clinical Communication & End-of-Life Care, School of Medicine, Deakin University

^5^ Department of Palliative Care, Calvary Mater, Newcastle, New South Wales

^6^ School of Medicine and Public Health, The University of Newcastle, New South Wales

^7^ Flinders University, College of Medicine and Public Health, Adelaide, South Australia

**Supplementary Figure 1**: number of participants by randomised arm of study continuing to take the intervention at each time point when comparing megestrol, dexamethasone and placebo for people with advanced cancer and poor appetite.


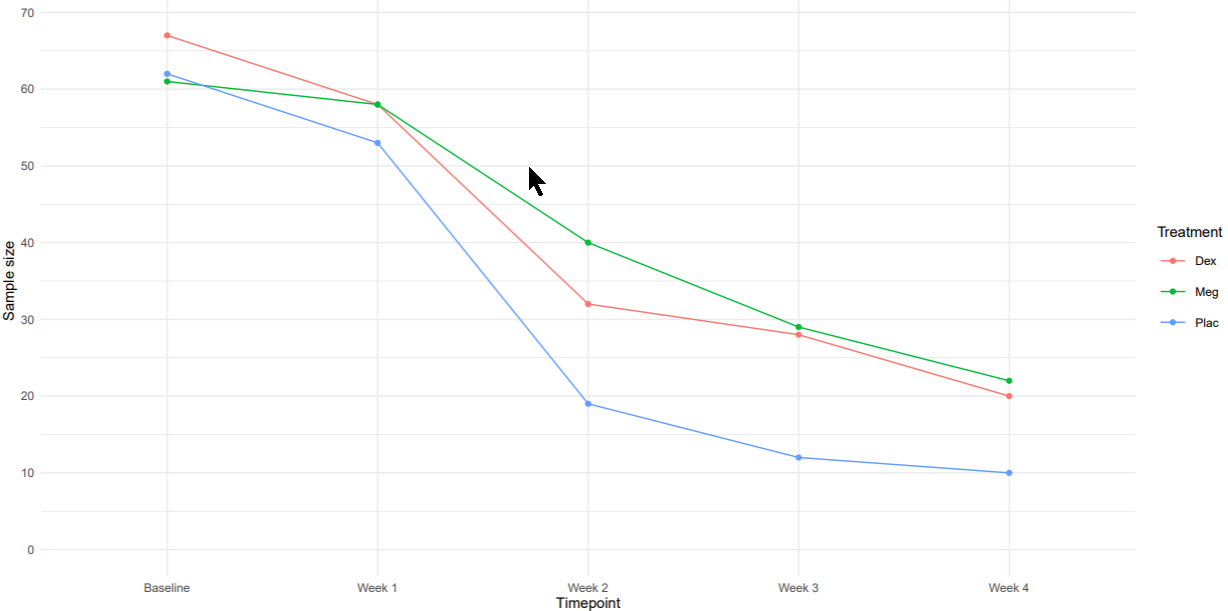


**Supplementary Figure 2**: Distribution of appetite scores on a 0-10 numerical rating scale (NRS; 0 = no appetite; 10 = best possible appetite) in a randomised trial comparing dexamethasone, megestrol and placebo.


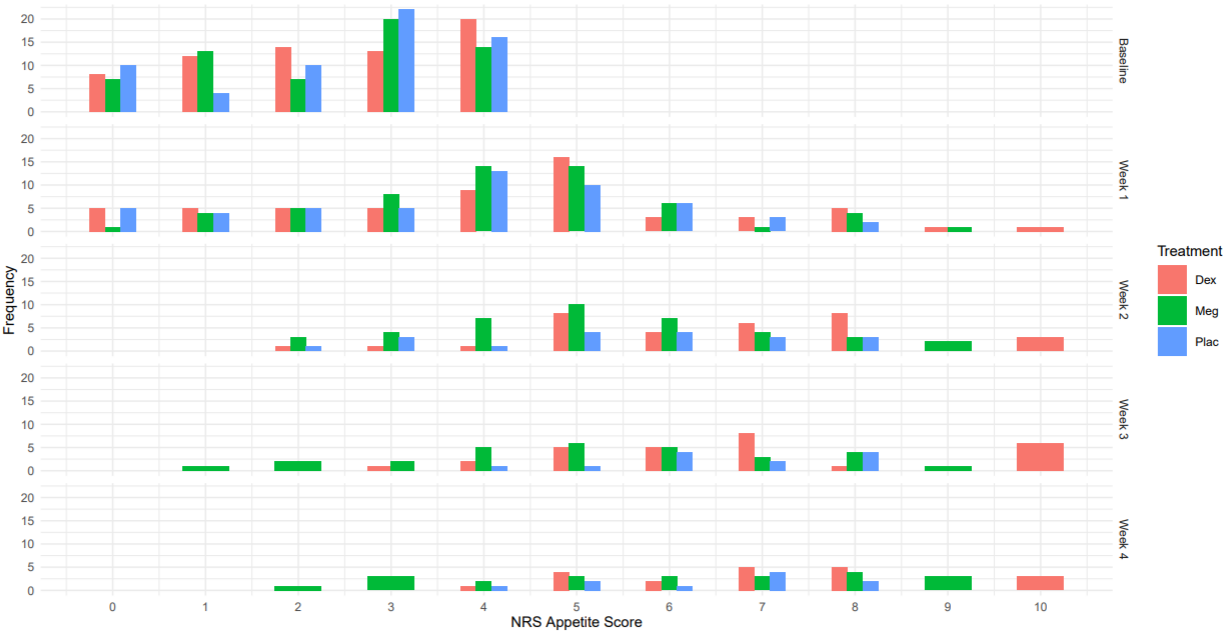


.
